# Competitive Transmission of Carbapenem-Resistant Klebsiella pneumoniae in a Newly Opened Intensive Care Unit

**DOI:** 10.1128/msystems.00799-22

**Published:** 2022-11-29

**Authors:** Ya Hu, Hui Zhang, Li Wei, Yu Feng, Hongxia Wen, Jingwen Li, Zhihui Zhang, Yongqiang Yang, Robert A. Moran, Alan McNally, Zhiyong Zong

**Affiliations:** a Center of Infectious Diseases, West China Hospital, Sichuan University, Chengdu, People’s Republic of China; b Department of Infection Control, West China Hospital, Sichuan University, Chengdu, People’s Republic of China; c Center for Pathogen Research, West China Hospital, Sichuan University, Chengdu, People’s Republic of China; d Neurogical ICU, West China Hospital, Sichuan University, Chengdu, People’s Republic of China; e Institute of Microbiology and Infection, College of Medical and Dental Sciences, University of Birmingham, Birmingham, UK; Marquette University

**Keywords:** *Klebsiella pneumoniae*, CRKP, transmission, intensive care unit, carbapenemases, carbapenem resistance, hospital environment, contamination

## Abstract

We conducted a 6-month prospective study in a newly opened ICU for high-resolution tracking of carbapenem-resistant Klebsiella pneumoniae (CRKP) through environmental surveillance, patient screening, and genome sequencing. Among all ICU patients (*n* = 348) screened, 3.5% carried CRKP on admission and 16.3% acquired CRKP thereafter. CRKP was not detected in the environment until 10 weeks and was then isolated from 98 of 2,989 environmental samples (3.3%). The first CRKP isolate from rectal swabs (*n* = 37) and the first clinical isolate (*n* = 8) of each patient as well as the 98 isolates from environmental were subjected to whole-genome sequencing. The 143 CRKP isolates from patients and environment samples were assigned to four sequence types, with ST11 dominating (95.8%) and further divided into 14 clones, suggesting introduction of multiple clones. Subsequent CRKP transmission was complex and dynamic with 10 clones found in multiple patients and seven also detected in the environment. Two particular ST11 clones caused extensive (≥5 rooms) and persistent (≥10 weeks) environmental contamination. Both clones were associated with patients who carried CRKP throughout their prolonged ICU stay. Such “super-contaminators” are a priority for isolation and environmental surveillance.

**IMPORTANCE** Carbapenem-resistant Klebsiella pneumoniae (CRKP) is a global challenge for human health. In health care settings, patients have frequent interactions with other patients and the environment, rendering challenges for untangling the introduction and transmission of CRKP. We conducted a prospective surveillance study in a newly opened ICU for high-resolution tracking of CRKP. Our study demonstrated the dynamic, complicated transmission of CRKP and has important findings that may help to curb its spread in health care settings. First, compliance with basic measures such as routine environment cleaning and postdischarge terminal cleaning is needed to minimize the environmental contamination-driven spread. Second, active screening could demonstrate the scale of the problem, and room transfer of patients with CRKP should be prohibited whenever possible. Third, the priority for single-room isolation should be given to patients with prolonged carriage of CRKP, especially in resource-limited settings. Good infection control practice lays a foundation for tackling multidrug-resistant organisms like CRKP.

## INTRODUCTION

Klebsiella pneumoniae is a leading cause of infections among intensive care unit (ICU) patients (1, 2). Carbapenems are important first-line antimicrobial agents used to treat severe infections caused by K. pneumoniae. However, carbapenem-resistant K. pneumoniae (CRKP) has emerged as a severe threat globally, associated with increased lengths of hospital stay and high mortality in patients (3), particularly those in ICUs (4–6). ICUs provide critical support for patients suffering from life-threatening conditions. This support involves extensive implementation of invasive medical devices such as ventilators and catheters. Such invasive measures expose ICU patients to pathogens carried by roommates and present in the hospital environment (7). As there are frequent interactions among patients and between patients and the environment in ICUs, untangling the introduction and transmission of CRKP is challenging.

We performed this study in a newly opened 21-bed neurological ICU at a major hospital in Chengdu, Sichuan Province, China, aiming to track the introduction and transmission of CRKP via epidemiological and genomic investigation. Sichuan is a developing region with a population of 83.67 million and a gross domestic product (GDP) per capita of $9,112 USD in 2020. Province-wide antimicrobial resistance surveillance has revealed that among 30,678 K. pneumoniae clinical isolates, the carbapenem-nonsusceptible rate was 7.7% in 2020 (8). We followed all patients admitted to the ICU for 27 weeks. CRKP recovered from patients and environmental samples were subjected to whole-genome sequencing. We revealed extensive, dynamic, and complicated transmission of multiple CRKP clones in the ICU with distinct patterns. Our data highlight the importance of the basic infection control measures such as daily routine environmental cleaning to control CRKP and demonstrate the priority of isolating patients with prolonged carriage of CRKP in resource-limited settings.

## RESULTS

### Many patients acquired CRKP in the ICU but few developed infections.

**FIG 1:**
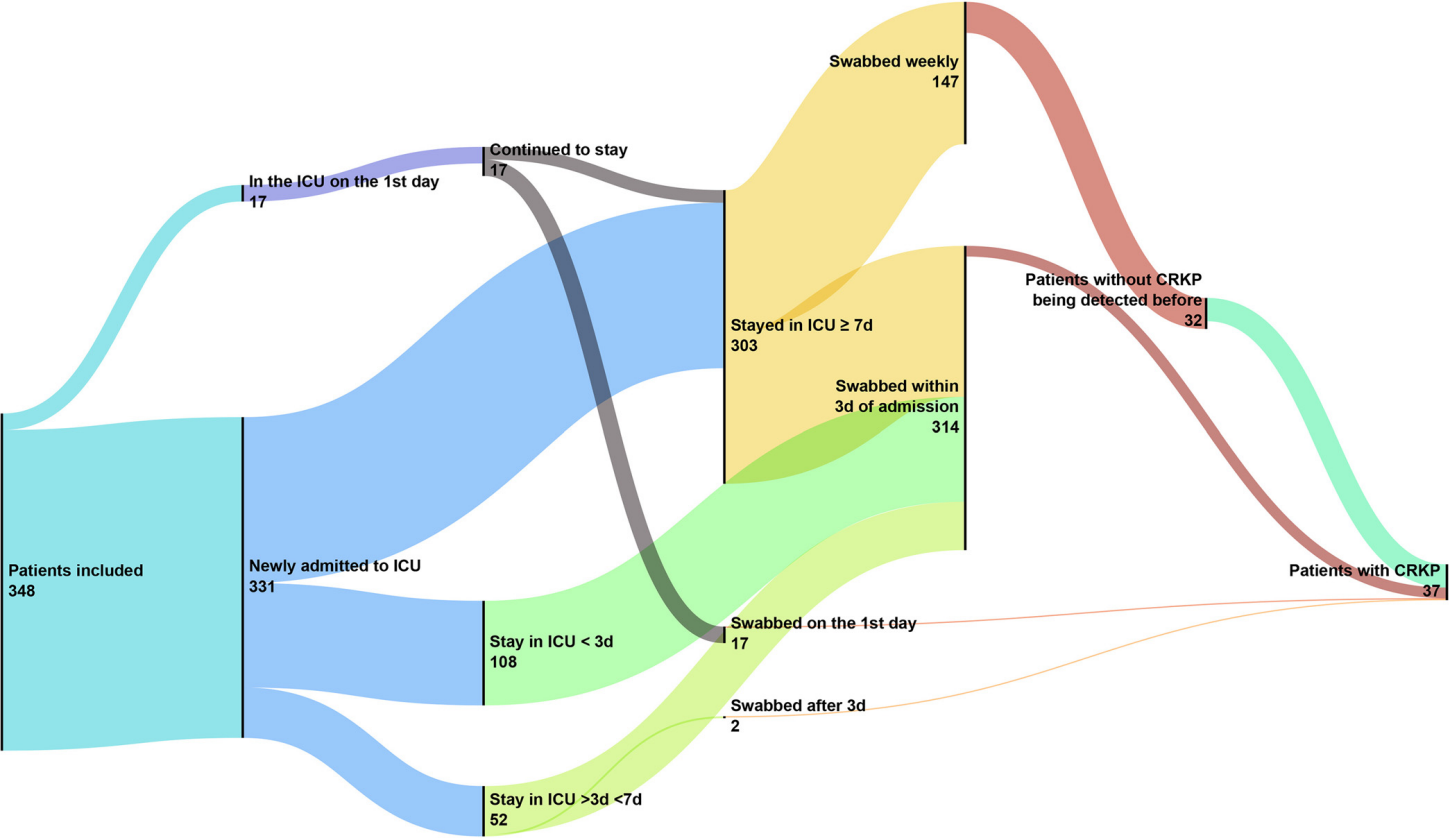
Flow chart of this study. During the study period, 348 patients were included. For each patient, the first CRKP isolate from rectal swabs and if the patient had infections, the first one from clinical samples were subjected to whole-genome sequencing; otherwise, isolates from the same patient were regarded as likely duplicated. This Sankey plot was made with RAWGraphs 2.0 (https://app.rawgraphs.io/).

**FIG 2:**
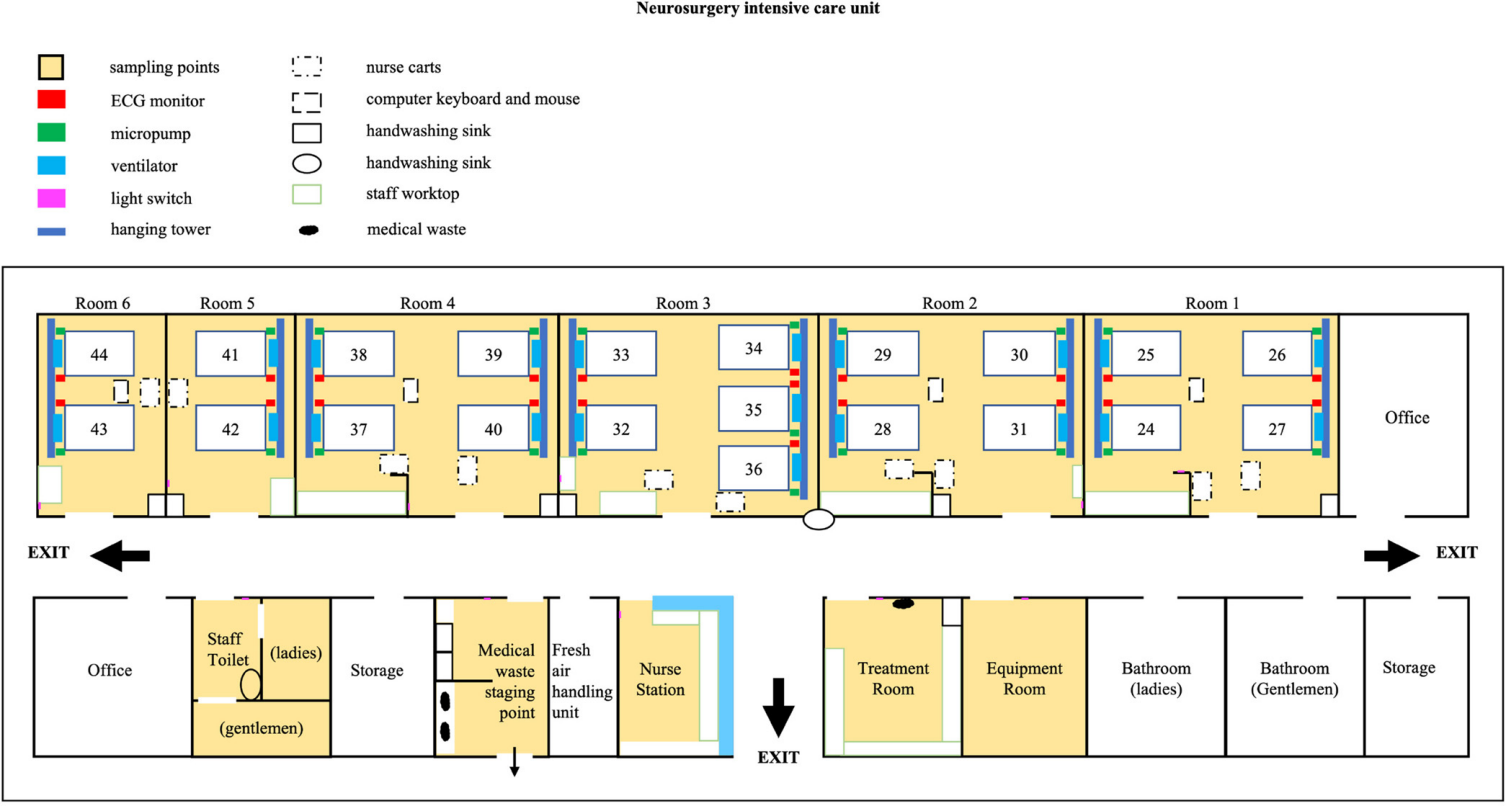
The ICU Plan. There are 6 rooms with 21 beds comprising three four-bed rooms (room 1, 2, and 4), two two-bed rooms (Rrom 5 and 6), and one five-bed room (room 3). Sampling sites are indicated in yellow.

A total of 348 patients were included in the study with the study flow chart shown by Fig. 1 and the ICU plan shown in Fig. 2. The cohort included 17 patients who were in the ICU on the first day of the study and 331 patients admitted to the ICU thereafter (Fig. 1). Rectal swabs were collected from all 17 patients on the first day of the study and CRKP was only isolated from one (P8).

Rectal swabs were collected within 3 days of admission from 316 of the 331 patients (95.5%) admitted to the ICU over the course of this study. CRKP was isolated from 11 of these 316 samples, corresponding to a 3.5% CRKP carriage rate on admission. In addition, two patients missed the swabs within 3 days after admission but were swabbed on the fourth day, and one was CRKP-positive. Over the course of the study 171 patients stayed in the ICU for at least 7 days, and 147 (86.0%, 147/171) were swabbed weekly until they were discharged. Of those 147 patients, 32 (21.8%, 32/147) yielded CRKP from at least one swab, 24 of which had been CRKP-negative on admission. The CRKP acquisition rate was therefore 16.3% (24/147).

During the study period, 8 patients had CRKP recovered from clinical samples, including sputum (*n* = 4; P108, P130, P270, and P319), urine (*n* = 2; P180 and P216), and pus (*n* = 2; P85 and P267). The CRKP infection rate during the study period was 2.3% (8/348). All of the 8 patients who were infected by CRKP had produced CRKP from rectal swabs collected prior to the CRKP-positive clinical samples; 5 from swabs on admission and 3 from weekly follow-up swabs.

### Extensive environmental surveillance revealed relatively common CRKP contamination of sinks, hanging towers, light switches, and bedrails.

The ICU underwent thorough clean and disinfection prior to opening. This study began 10 days after opening and environmental sampling on the first day of study did not yield CRKP. CRKP was not detected in the ICU environment until week 9. From week 9, CRKP isolates were gradually identified from the ICU environment. A total of 2,989 environmental samples were collected during the study period from the sampling sites (Table 1). CRKP was isolated from 98 samples corresponding to a 3.3% (98/2,989) positive rate, including 55 from patients’ immediate surroundings, 17 from shared areas in patient rooms, and 24 from areas outside patient rooms (Table 1).

**TABLE 1:** NICU environment sampling results

Sampling area	Sampling site	Samples, n	CRKP-positive samples, n (%)
Bed surroundings	Bed rails	290	11 (3.8%)
	Hanging tower	292	16 (5.5%)
	ECG monitor	292	4 (1.4%)
	Stethoscope	283	6 (2.1%)
	Ventilator	270	7 (2.6%)
	Air pump	250	2 (0.1%)
	Infusion pump	269	9 (3.3%)
Shared area in patient rooms	Computer keyboard and mouse	140	4 (2.9%)
	Nurse cart	138	4 (2.9%)
	Light switch	83	3 (3.6%)
	Sink: drain	83	3 (3.6%)
	Sink: faucet	83	2 (2.4%)
	Sink: top surface	83	1 (1.2%)
Common area outside patient room	Computer keyboard and mouse	55	3 (5.5%)
	Printer	36	3 (8.3%)
	Barcode scanner and printer	28	0 (0.0%)
	Instrument cabinet	24	0 (0.0%)
	Liquid storage cabinet	12	0 (0.0%)
	Treatment tabletop	12	1 (8.3%)
	Storage rack	12	1 (8.3%)
	Door handle	36	0 (0.0%)
	Light switch	43	0 (0.0%)
	Sink: drain	48	8 (16.7%)
	Sink: faucet	48	2 (4.2%)
	Sink: top surface	48	7 (14.6%)
	Sink: inner surface of overflow	24	1 (4.2%)
	Ventilator	2	0 (0.0%)
	Vibration sputum excretory system	5	0 (0.0%)
Total		2,989	98 (3.3%)

The total number of samples collected for each sampling type (e.g., bed rails, sink drains, and light switches) varied according to the number of sampling sites (Table 1). For patient bed surroundings, hanging towers were the most contaminated site with a 5.5% CRKP positive rate followed by 4.1% of bed rails (Table 1). For shared area in patient rooms, both light switches and sink drains had the highest CRKP positive rate (3.6%). For common areas outside patient rooms, sinks were the most contaminated sites with a 16.7% CRKP positive rate for drains and 14.6% for top surfaces (Table 1). Of note, CRKP was never detected from barcode scanners and printers, door handles, light switches at the common area, instrument cabinet, liquid storage cabinet in the treatment room, and ventilator, vibration sputum excretory system in the equipment room during the 27-week study period (Table 1).

### CRKP isolates belonged to multiple sequence types but sequence type (ST) 11 KL64 dominated.

For each patient, the first CRKP isolate from rectal swabs and, where present, the first clinical isolate were regarded unique and were included for genome sequencing, while further isolates from the same patient were likely duplicates and were not included. As described above, there were a total of 143 unique CRKP isolates recovered in this study (Data set S1). These 143 isolates comprised 37 from rectal swabs (1 from a patient sampled on the first day of this study, 11 from patients detected on admission, 1 from a patient detected on the fourth day after admission, and 24 from patients sampled on weekly follow-up occasions; Fig. 1), 8 from clinical samples, and 98 from the environment. All of the 143 isolates were resistant to meropenem (Meropenem minimum inhibitor concentration [MIC], 8 to 512 mg/L; Data set S1) and were subjected to whole-genome sequencing. The vast majority of the 143 isolates belonged to ST11 (*n* = 133, 93.0%), with the remainder ST307 (n = 4, all 4 of capsular locus [KL]102), ST22 (*n* = 1, KL9), or ST37 (*n* = 1, KL118). Four isolates (all KL64) could not be assigned to a known sequence type due to the absence of the *mdh* allele. The other six MLST alleles in these isolates were identical to those of ST11, while their *mdh* alleles were found to be interrupted by a copy of the insertion sequence IS*26*, flanked by the 8-bp target site duplication (TSD; CTGAAAAA). When the IS*26* and one copy of the TSD were removed *in silico* the uninterrupted *mdh* was identical to that of ST11. Therefore, we regarded the four isolates as ST11, bringing the total number to 137, representing 95.8% of all 143 CRKP isolates. Of the 137 ST11 CRKP isolates, 124 were capsular type KL64 (90.5%) and 13 were KL47 (9.5%). Notably, among the 24 patients who acquired CRKP in the ICU, 22 acquired ST11 CRKP (19 KL64; 3 KL47) while the remaining two acquired ST307 (Data set S1).

10.1128/msystems.00799-22.2 DATA SET S1 CRKP strains in this study. Download Data Set S1, XLSX file, 0.02 MB. Copyright © 2022 Hu et al. 2022 Hu et al. https://creativecommons.org/licenses/by/4.0/ This content is distributed under the terms of the Creative Commons Attribution 4.0 International license.

All of the ST11 isolates carried the carbapenemase-encoding gene *bla*_KPC-2_ and four of them (all of KL64) also had *bla*_NDM-1_ (Data set S1). The two ST37 isolates and the ST22 one had *bla*_NDM-5_. Three ST307 isolates carried *bla*_NDM-1_, while the remaining ST307 isolate had no known carbapenemase-encoding genes but carried the extended-spectrum β-lactamase (ESBL) gene *bla*_CTX-M-15_ and had a truncation on OmpK36 (Data set S1). The combination of ESBL production and truncation or point mutation of OmpK36 is known to confer carbapenem resistance (9).

### ST11 CRKP isolates were assigned to a diversity of clones.

We focused on isolates of the dominant ST11 type to study CRKP transmission in the ICU. For all ST11 genomes in this study, the estimated nucleotide substitution rate per genome per year was 3.06 to 9.15 (mean 5.45). The maximum estimated nucleotide substitution rate per genome in a half year would be 4.58 (about 5). We therefore chose a threshold of 5 high-quality core-genome single-nucleotide polymorphisms (SNPs) for clone delineation, and any two isolates that differed by ≤5 SNPs were assigned to the same clone.

**FIG 3:**
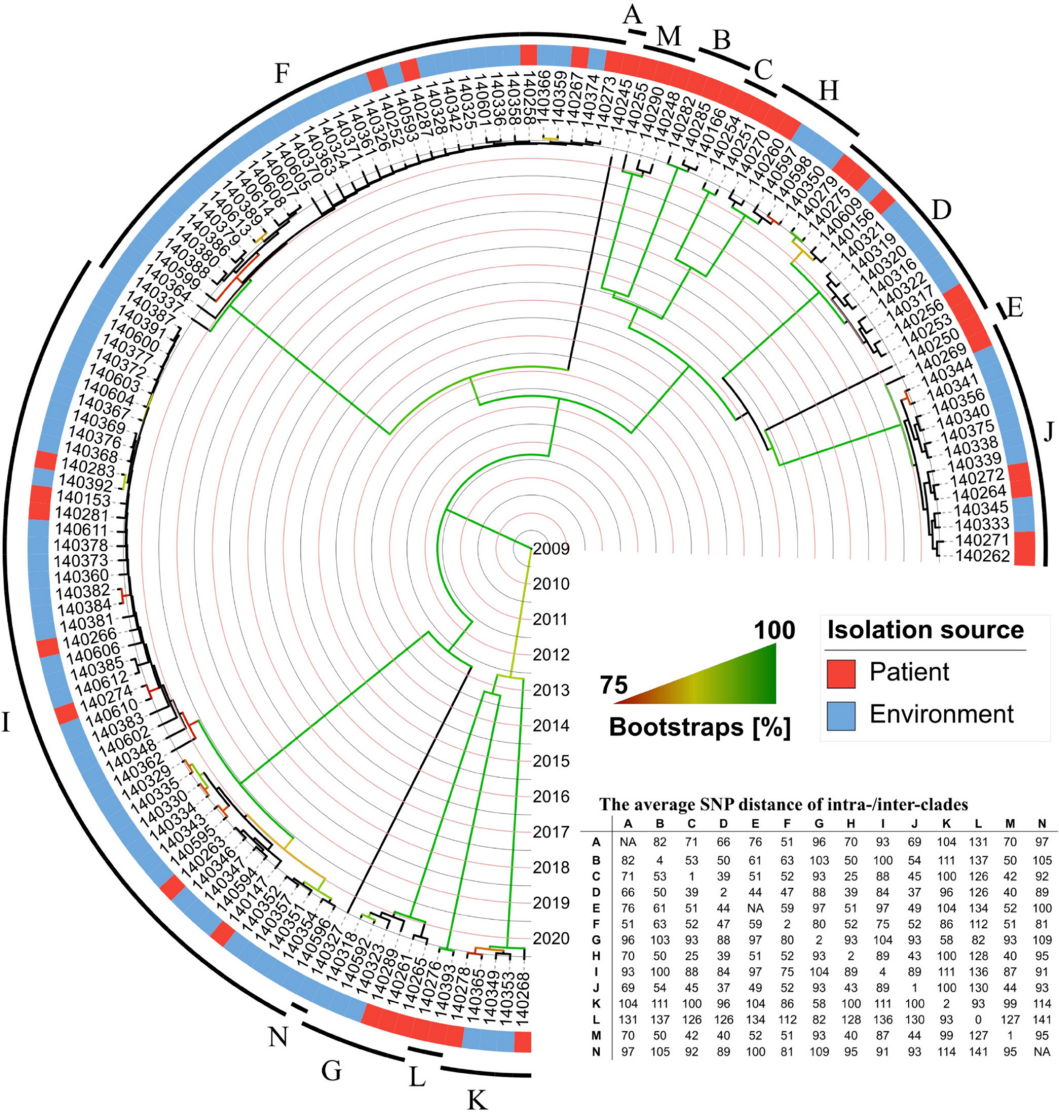
A phylogenetic tree of 137 ST11 CRKP strains. Isolate numbers were shown and capital letters A to N represent the clone assignation. The average SNP differences of intra- and interclone are indicated on the embedded table, while the exact ranges of these SNP differences are shown in Table S1. -, there are no interclone SNP differences for clones comprising a single isolate.

The 137 ST11 CRKP isolates were assigned to 14 clones, including 11 that were KL64 (clone A to F, I, J, L, M, and N) and 3 that were KL47 (G, L, and K) (Fig. 3). There were 0 to 13 SNPs between isolates of each clone but 23 to 141 SNPs between any two clones (Table S1). The 8 isolates from clinical samples all belonged to ST11 but were assigned to 7 different clones (one isolate for each of clone B, D, F, G, L, and M and two for clone I). Of note, 7 of the 8 clinical isolates belonged to the same clone as the corresponding isolates recovered from rectal swabs of the same patient. However, patient P130 had a clone G clinical isolate and a clone F isolate from rectal swab, both of which were recovered from the sample or swab collected on the same day of admission. This suggests coexistence of two strains of ST11 CRKP in a single patient and cointroduction in the ICU.

10.1128/msystems.00799-22.1 TABLE S1 The intra- and interclone SNP numbers of ST11 CRKP strains. Download Table S1, PDF file, 0.02 MB. Copyright © 2022 Hu et al. 2022 Hu et al. https://creativecommons.org/licenses/by/4.0/ This content is distributed under the terms of the Creative Commons Attribution 4.0 International license.

### CRKP was introduced to the ICU on multiple occasions.

The 14 ST11 clones exhibited difference in their origins, association with patients, possible transmissions between patients, and extent of environmental contamination (Table 2, Data set S2 and Data set S3). Nine clones (B, D to H, J, L, and M) were introduced by patients as the first strains of these clones were isolated from patient rectal swabs collected on the day of admission (8 clones; B, D, F, G, H, J, L, and M) or within 2 days of admission (clone E) (Data set S3). In contrast, the exact origin of the remaining five clones (A, C, I, K, and N) remained unclear. Clone A was only detected in a rectal swab collected on the first day of the study, from a patient who had been in the ICU for 6 days (Data set S3). The first isolates of clone C, I, and K in the ICU detected from rectal swabs or clinical samples collected from patients after 2 days of admission, while clone N was only detected in an environment sample at week 13 of this study (Data set S3). Nonetheless, as this ICU was newly open, several rounds of environmental sampling since the study start (10 days after opening of the ICU) did not grow CRKP; it was very likely that these clones were also introduced rather than preexisting in the ICU.

10.1128/msystems.00799-22.3 DATA SET S2 Environmental sampling and corresponding CRKP strains. Download Data Set S2, XLSX file, 0.01 MB. Copyright © 2022 Hu et al. 2022 Hu et al. https://creativecommons.org/licenses/by/4.0/ This content is distributed under the terms of the Creative Commons Attribution 4.0 International license.

10.1128/msystems.00799-22.4 DATA SET S3 Patients and their CRKP strains. Download Data Set S3, XLSX file, 0.02 MB. Copyright © 2022 Hu et al. 2022 Hu et al. https://creativecommons.org/licenses/by/4.0/ This content is distributed under the terms of the Creative Commons Attribution 4.0 International license.

**TABLE 2:** The distribution of clones in patients

Clone^*a*^	Patients^*b*^				Environmental contamination^*c*^
	Total no.	Introduction	Acquisition	Infection	
A	1	P8	-	-	-
B	2	P85	P126	P85	-
C	2	-^*e*^	P102, P113	-	-
D	4	P129	P90, P256, P267	P267	Yes
E	1	P116	-	-	-
F	4	P130	P108, P191, P243	P108	Yes
G	3	P130, P209	P159	P130	Yes
H	2	P117, P234^*d*^	-	-	Yes
I	5	P216	P180, P215, P280, P298	P180, P216	Yes
J	5	P193	P192, P221, P236, P237	-	Yes
K	2	-	P214, P232	-	Yes
L	1	P270	-	P270	-
M	2	P319	P328	P319	-
N	0	-	-	-	Yes
ST307	2	-	P262, P308	-	Yes

a Clones belonging to the KL47 capsular type (G, L, and K) are underlined. Other clones belonged to KL64.

b All patients listed here had at least a rectal swab positive to CRKP. Details of the introduction, acquisition, and infection of each patient are available in Supplementary Data set S3.

c Details of environmental contamination are available in Supplementary Data set S2.

d Introduction of clone H in P234 was hypothesized as no rectal swabs were collected within three days of admission.

e Dash indicates ‘none’.

### Both intra- and interroom transmission between patients were identified, and most CRKP clones were involved in limited transmission.

**FIG 4:**
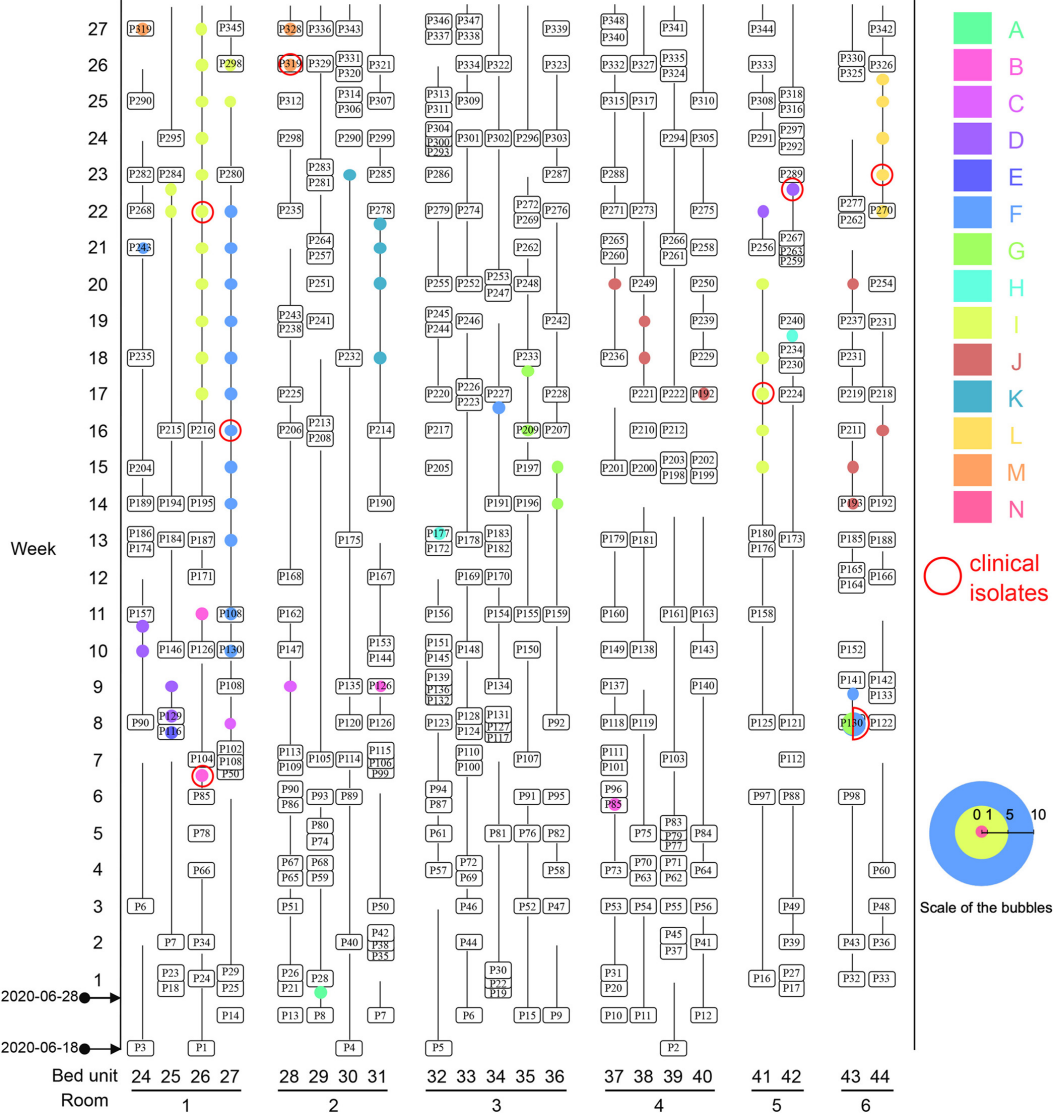
Bubble diagram of patients with ST11 CRKP. The horizontal axis illustrates the bed and room number of the patients, while the vertical axis indicates the weeks. Patients are labeled and the longitudinal lines connecting patient labels exhibit the corresponding length of stay in ICU. Any two isolates that differed by ≤5 single nucleotide polymorphisms were assigned to the same clone and CRKP of different clones are marked by circles in colors. Clinical isolates are highlighted with a red circle of the bubbles. The radium of each bubble (not the red circle) is proportional to the number of isolates from the same patient in the same week and the scale of the radium is shown. Bubbles indicate that the isolate was recovered at the time point and refers to the present patient if the bubble is on the patients’ identifier or the prior patient if the bubble is on the line with connecting the prior patient.

Of the 14 ST11 clones, 13 were isolated from patient rectal swabs and/or clinical samples. Of the 13 patient-associated clones, three (A, E, and L) were only found in a single patient each while five (B, C, H, K, and M) were seen in two patients each (Fig. 4 and Data set S3).

Among the five clones seen in two patients each, clone K was seen in two patients on different beds in the same room, while clone M was seen in two patients who stayed on the same bed consecutively (Data set S3). This suggests different routes of intraroom interpatient transmission: cohabitation of a room for clone K and longitudinal use of the same bed for clone M. In contrast, clones B, C, and H were isolated from patients in different rooms in different weeks of the study and were never detected in environmental samples (Data set S2). The lack of environmental isolates of clones B, C, and H suggest that these clones did not establish and persist in environmental reservoirs that facilitated transmission. The presence of these clones in multiple patients and rooms might therefore be the result of transmission via an undetermined route, or of multiple introductions to the ICU.

Clone G was isolated from three patients. Two of these patients were carrying clone G on admission, P130 in week 8 (room 6) and P209 in week 17 (room 3) (Data set S3). It therefore appears that clone G was introduced to the ICU independently on two occasions. After its initial introduction with P130, clone G was isolated from two environmental sites in room 1 and from the sink in the shared medical waste staging area in week 11 (Data set S2). P159 who was CRKP-negative on admission to room 3 in week 12 later produced clone G from a rectal swab collected in week 14. P159 was admitted 2 days after P130 was discharged, and clone G had not been isolated from room 3 prior to P159’s admission. How P159 acquired clone G is therefore unclear, but the detection of this clone in a common-area sink suggests that it persisted in an environmental reservoir to facilitate transmission.

### Several clones caused extensive and persistent environmental contamination.

Among the 22 patients who acquired ST11 CRKP in the ICU, 14 (63.6%) acquired clones D, F, I, and J (Data set S3). Each of these clones was isolated from more than three patients: two or more patients in the same room and from patients in different rooms (Fig. 4).

Clone D was seen in four patients in two rooms, but its appearances in these rooms were separated by a 10-week interval (Data set S3). Clone D was first introduced to room 1 with P129 in week 8. and was acquired by their roommate, P90, who had been CRKP-negative on admission in week 6 but produced clone D in week 10. Clone D was also isolated from environmental sites in room 1 in weeks 9 and 11. By week 11, P129 and P90 had been discharged, and clone D was not isolated again for 10 consecutive weeks. P256 and P267, both CRKP-negative on admission to room 5 in week 21, produced clone D from rectal swabs in weeks 22 and 23. As clone D had not been isolated from any of the five environmental sampling rounds between its appearances in room 1 and 5, persistence in the ICU seems unlikely. However, if it was introduced to the ICU a second time, the source of this introduction has not been identified.

Clone J was seen in five patients in two rooms: three (P192, P193, and P237) in room 6 and two (P221 and P236) in room 4 (Data set S3). Clone J was introduced to the ICU by P193 at week 14, who was discharged at week 16. Two patients in room 6, P192 and P237, acquired this clone at week 16 and 20, respectively. Of note, P237 had no overlapped stay in room 6 with P192 and P193 but had stayed in the bed unit (bed 43) used for P193, suggesting the acquisition of this clone by P237 driven by sharing a common bed. P192 was transferred from room 6 to room 4 at week 16. After the transfer, two roommates, P221 and P236, who overlapped with P192 in room 4 (but at different beds), acquired clone J at week 18 and 20, respectively (Fig. 4). This suggests an interroom transmission event driven by patient transfer. An infusion pump and a computer keyboard in room 4 were positive to this clone at week 15 and 17, respectively (Data set S2), and the environmental contamination may therefore facilitate the intraroom transmissions of clone J.

### The extensive and persistent environment contamination of clone F and I was largely driven by patients with continuous carriage and prolonged ICU stay.

Clones F and I exhibited extensive environmental contamination and persistence. These clones were isolated continuously from their first appearances in the ICU to the end of this study and were present in five of the six patient rooms and three (clone F) or six (clone I) different sites in the common areas (Data set S2 and S3). Both clones were associated with patients who stayed in the ICU for prolonged periods.

**FIG 5:**
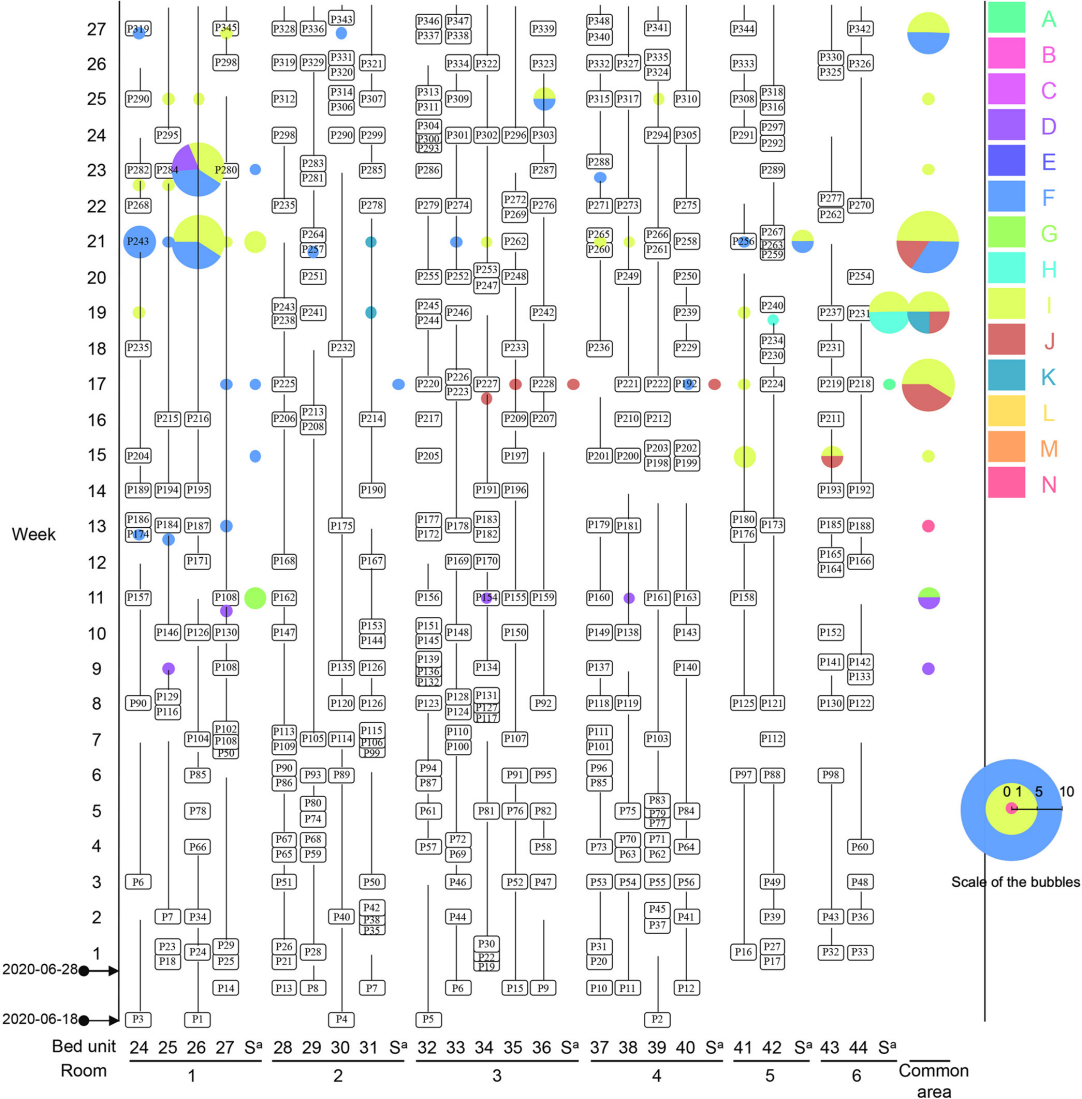
Bubble diagram of environmental contamination of ST11 CRKP. The horizontal axis illustrates the bed and room number, shared areas in patient rooms abbreviated as S^a^, and common area outside patient rooms. The vertical axis indicates the weeks. Patients are labeled and the longitudinal lines connecting patient labels exhibit the corresponding length of stay in ICU. CRKP of different clones are marked by bubbles in colors. The radium of each bubble is proportional to the number of isolates from the same environmental site in the same sampling round.

Clone F first appeared in a P130 rectal swab in room 6 on admission in week 8. P130 was transferred to room 1 in week 9 and discharged in week 11 (Data set S3). On the same day that P130 was discharged, P108 was transferred to the bed unit that P130 had occupied. In week 13, clone F was isolated from a P108 rectal swab. P108 rectal swabs were CRKP-positive for the next 10 consecutive weeks. From this point, clone F was isolated from multiple environmental samples in patient rooms and common areas until the end of the study, including from computer keyboards at the nurse station (Fig. 5 and Data set S2). Patient P243 in room 1 and P191 in room 3 acquired clone F in week 21 and 17, respectively, suggesting both intraroom (room 1) and interroom transmission (to room 3). As P191 did not share a room with other patients carrying clone F, it seems likely that extensive environmental contamination across multiple rooms and the common area led to their acquisition of clone F.

Clone I was first isolated in week 15, from patient P180 in room 5. P180 rectal swabs were CRKP-positive for the following five consecutive weeks, until P180 was discharged in week 20. Patient P216 in room 1 was found to have clone I in a rectal swab collected 1 day after their admission to the ICU in week 17, 11 days after the first detection of this clone in P180. P180 still had CRKP-positive swabs when P216 was admitted. It is possible that P216 acquired clone I in the ICU. P216 had CRKP-positive rectal swabs for 10 consecutive weeks to the end of this study. Clone I was first found in the environment in week 15, the same week when it had first been isolated from P180 and persisted to the end of this study. Clone I was detected in computer keyboards or printers at the nurse station at week 15 and 17 (Data set S2). Environmental contamination could lead to the transmission between P180 and P216 in different rooms. Clone I was also acquired by patients P215, P280, and P298 who hospitalized in the same room as P216 and had overlapping stay period with P216, suggesting the predominate intraroom transmission of this clone by room sharing.

## DISCUSSION

A newly opened ICU provided us with a unique opportunity to prospectively investigate the introduction and subsequent transmission of CRKP to a working ICU environment. The combination of whole-genome sequencing and patient information revealed highly dynamic, complicated introduction, transmission, and competition of CRKP strains in this newly open ICU. There were continuous introductions of CRKP, which could be due to new introduction of various clones or repeated introduction of the same clone to the ICU. This highlights the importance of active screening on admission and the application of whole-genome sequencing in tracking transmission in high-resolution.

Many patients acquired CRKP in ICU, which were largely driven by the transmission of several particular clones. Consistent with previous studies of others and ours in China (7, 10–12), ST11 is the dominant type of CRKP and KL64 exceeds KL47 as the major capsular type in China (13). CRKP clones of the same sequence and capsular types are similar in genome sequences, may be introduced concurrently, and spread simultaneously but have quite different destinies in the ICU. Some clones disappeared shortly after introduction without causing transmission and some others spread to only one patient, while several clones were able to cause extensive transmission among multiple patients across different rooms. As revealed in this study, the intraroom spread of the same clone between patients could be due to their longitudinal stay in the same bed or simultaneous stay in the same room. The interroom spread was associated with patient transfer and extensive environmental contamination.

Environmental contamination by CRKP is common as described previously (7, 14). The isolation of CRKP clones from common areas outside patient rooms, such as staff computer keyboards and printers, likely reflects noncompliance to hand hygiene protocols by health care workers. It appears that most CRKP clones remained only transiently in the ICU environmental as they were only detected in a single sampling round and disappeared when their carrier patients were discharged. This supports the effectiveness of daily cleaning practices. In contrast, two clones caused extensive and persistent contamination, which was largely associated with individual patients with continuous CRKP carriage and prolonged ICU stay of more than 6 weeks. The continuous carriage prolongs the shedding of CRKP into the environment causing persistent contaminations, while the long ICU stay increases the interaction with other patients and with health care workers to expand the contamination and facilitate spreading of bacterial strains. These index patients could serve as “super-contaminators” driving extensive and persistent environmental contamination amplifying the spread of their carried CRKP clones. This suggests that the “super-contaminator” should be the priority for isolation. Unfortunately, there are no single rooms in this ICU, but the two-patient rooms could be an option to isolate such CRKP-carrying patients with a predicted ICU stay of 6 weeks or longer to minimize interpatient transmission. Ideally, the two-patient rooms could be dedicated for such patients. Fortunately, the hospital administration has realized the problem of lacking single rooms in this ICU after being informed by the results of this study, and there is a plan to expand the ICU capacity in 2022 and 2023 and this ICU will be merged with an adjacent unit with available single rooms. Surveillance cultures of the environment consume much-needed resources, including manpower and require specialized approaches that usually need capacity-building in resource-limited settings (15). In such settings, the “super-contaminator” could be a priority of environment surveillance to guide effective cleaning and maximize the benefits of the use of limited resources in controlling CRKP.

The strengths of this study include the perspective nature, the newly open unit as the study place, the large number of environmental samples, and the combination of environmental surveillance, patient active screening, patient information, and high-resolution whole-genome analysis for strain tracking. We are aware of limitations of this study. First, this was a single-site study with intrinsic weaknesses such as restricted generality. It was also carried out in a single unit, which cannot capture transmissions outside this unit in the hospital. Second, due to the pragmatic consideration of our limited manpower, in particular during the COVID-19 pandemic, environmental sampling was performed once every 2 weeks. Such intervals could miss the contamination in the intervening period and therefore may not restore all transmissions associated with environmental contamination. Third, no patient visitors are allowed in this ICU, but we did not screen our health care workers for CRKP carriage and did not sample their hands and clothing to detect CRKP contaminations. Transmissions associated with health care workers therefore cannot be identified.

Despite the limitations, this study provides much-needed insights into CRKP transmission in the ICU and generated useful data to inform infection control. First, the contamination of common areas, the transient contamination by most clones, and transmission by longitudinal sharing the same bed highlight the paramount importance of complying with basic measures such as hand hygiene, routine environment cleaning, and terminal cleaning and disinfection after patient discharge. The frequent detections of CRKP in sinks and hanging towers could inform good cleaning practice. Second, room transfer of patients, in particular those with CRKP, should be prohibited whenever possible. Third, patients with continuous CRKP carriage and prolonged ICU stay are “super-contaminators” and should be the priority for isolation and environmental surveillance.

**Conclusion**. We performed a prospective study in a newly opened ICU by patient-active screening and environmental surveillance for tracking CRKP during a period of 6 months. We identified the frequent introduction of multiple ST11 CRKP clones and sometimes repeated introduction of the same clone along with patients. We demonstrated that the highly dynamic and complicated transmission, both intra- and interroom, of and simultaneous competition between the clones with several particular ones being more successfully transmitted. We found that environmental contamination of CRKP is commonly transient but certain clones can cause extensive and persistent contamination, which is due to the continuous carriage by patients with prolonged ICU stay of more than 6 weeks as “super-contaminators.” Good cleaning practice and prohibiting room transfer of patients are critical to curb CRKP transmission, while “super-contaminators” should be given the priority for isolation and environmental surveillance.

## MATERIALS AND METHODS

### Study design.

This was a prospective investigation study that was conducted for 27 weeks between June 28, 2020, and December 31, 2020, in a newly opened neurological ICU at a tertiary teaching hospital. The ICU was opened on June 18, 2020, and this study began 10 days later. Week numbers referred to in this text were assigned consecutively from the start of the study; for instance, week 1 refers to the week from June 28 to July 4, 2020. This study was approved by the Ethical Committee of West China Hospital with informed consent being waived.

### Patients and samples.

Patients were numbered according to their order of admission to the ICU. Patient identifiers consisted of a P representing the word “patient” and the patient number; for instance, P1 refers to the first patient admitted to the ICU. Rectal swabs were collected from patients within 3 days of admission and then once weekly over the course of their ICU stay. Both admission and discharge in the following text refer to those to and from the ICU.

### Environmental surveillance.

The ICU has 21 beds in six patient rooms, including two-bed (room 5 and 6), four-bed (room 1, 2, and 4), and five-bed (room 3, Fig. 2) configurations. Environmental sampling was performed every 2 weeks. Sampling sites included patient rooms and common areas outside patient rooms, including the nurse station, medical waste staging point (temporary storage), equipment room, treatment room, and staff toilet.

In patient rooms, we sampled multiple sites of each bed unit, including the entire surface of bedrails at four sides (right, left, head, and foot), the stethoscope, bed air pump buttons, and ventilator button panels, cardiovascular monitors, infusion pumps, and a 1,200 cm^2^ (30 cm × 40 cm) hanging tower surface. For the communal areas within patient rooms, we sampled the light switch, nurses’ carts plus the entire surface of cart drawer handles, and the entire surfaces of computer keyboards and mice. Each of the six rooms has a handwashing sink. All sinks, including faucets, the top surfaces, and drains were sampled.

In the nurse station, we sampled entire surfaces of computer keyboards and mouses, printers, and barcode scanners. The handwashing sink near the nurse station in the corridor was sampled as described above. In the medical waste staging point, we sampled entire surfaces of the light switch, storage racks, and sinks. For the equipment room, we sampled the entire surface of the light switch, the ventilator, and the vibration sputum excretory system. In the treatment room, we sampled the entire surface of the light switch, a 30 cm × 40 cm surface on the liquid storage cabinet, and the treatment tabletop. In the staff toilet, we sampled entire surfaces of the door handles, the light switch, and the two sinks.

Surfaces and sinks were sampled using sterile rayon swabs (Copan; Brescia, Italy) moistened with tryptic soy broth (TSB; Hopebio, Qiangdao, China). The swabs from surfaces were streaked onto Simmon’s Citrate agar plates (supplemented with 1% inositol) containing 2 mg/L meropenem to screen CRKP. Swabs from sinks were immediately placed into 15 mL sterile tubes containing 6 mL TSB after sampling, which were incubated at 37°C overnight and were centrifuged. Supernatants were discarded and cell pellets were resuspended in 1 mL TSB. A 50 μL suspension aliquot was streaked onto Simmon’s Citrate agar plates (supplemented with 1% inositol) containing 2 mg/L meropenem.

### Whole-genome sequencing and analysis.

For each patient, the first CRKP isolate from rectal swabs and, where present, the first clinical isolate was subjected to whole-genome sequencing; otherwise, isolates from the same patient were regarded as likely duplicates and were excluded from genome sequencing. Genomic DNA was prepared using the QIAamp DNA minikit (Qiagen; Hilden, Germany), and DNA libraries were prepared using the NEBNext Ultra II DNA Library Prep kit for Illumina platform (NEB; Ipswich, MA, USA). Genome sequencing was performed using the Illumina HiSeq X10 platform (Illumina; San Diego, CA, USA). To obtain a complete reference genome for mapping, a representative isolate, 140253, which was a ST11 KL64 strain from a rectal swab of patient (P129) collected on admission at week 8 in this study, was also subjected to long-read genome sequencing by a MinION Sequencer (Nanopore; Oxford, UK).

### Genome assembling and profiling.

Short reads were assembled into draft genomes using SPAdes v3.15.4 (16) in careful mode. The complete genome of strain 140253 was obtained by *de novo* hybrid assembly of both short and long reads using Unicycler v0.5.0 (17) and was polished with Pilon v1.24 (18). ST was determined using the assembled genomes to query the multilocus sequence typing database (https://bigsdb.pasteur.fr/klebsiella/). Capsule locus typing and identification of modifications (truncations and mutations) of outer membrane porins OmpK35 and OmpK36 were performed using Kleborate v2.2.0 (19). Acquired antimicrobial resistance genes were identified using ResFinder (http://genomicepidemiology.org/).

### SNP calling and phylogenetics.

SNPs among CRKP isolates of the same ST and KL type were called using Snippy v4.6.0 (https://github.com/tseemann/snippy) with default settings. A maximum-likelihood tree of ST11 CRKP was inferred from the pseudo whole-genome alignment generated by applying SNPs to the reference strain 140253 for each individual genome. The tree was inferred using RAxML v8.2.12 (20) under GTRGAMMA model and a 1,000-bootstrap test with masking recombination regions identified by Gubbins v3.2.1 (21) and plasmids. Two additional ST11 strains from our hospital in 2015, 095084 (KL47, accession CP027068) and 095649 (KL64, accession CP026585), representing the first of their capsular type isolated here, were additionally included into the phylogenetic tree construction to robustly root the phylogeny and to avoid the bias of elevated nucleotide substitution rate due to the short sampling time (22).

### Time-calibrated tree estimating.

The emergence time of the most recent common ancestor (MRCA) of each clone of ST11 CRKP and the average nucleotide substitution rate per genome per year for ST11 CRKPs in this study were estimated using BactDating v1.1.0 (23) under mixedcarc model with a 10^7^- iterations Markov Chain Monte Carlo (MCMC). The estimated nucleotide substitution rate per genome per year of ST11 CRKP in this study was also calculated using BactDating v1.1.0 (23). Having considered that transmission could occur in a cascade-like pattern, any two strains with SNP differences lower than the maximum estimated nucleotide substitution rate per genome in a half year were assigned to the same clone; otherwise, the strains were assigned to different clones.

### Antimicrobial susceptibility testing.

Meropenem MICs were determined using the broth microdilution method according to the guidelines of the Clinical and Laboratory Standards Institute (CLSI) (24). Escherichia coli strain ATCC 25922 was used as control.

### Data availability.

The draft genomes of the isolates in this collection have been deposited in NCBI under BioProject accession PRJNA839691. The complete genome sequence of strain 140253 has been deposited in GenBank under accession numbers CP097626 to CP097631.
