# Supplementary material for: Competitive Transmission of Carbapenem-Resistant Klebsiella pneumoniae in a Newly Opened Intensive Care Unit
Source: mSystems. 2022 Nov 29;7(6):e00799-22. doi: 10.1128/msystems.00799-22 (PMC9764986; doi:10.1128/msystems.00799-22)
Supplement: TABLE S1 [file msystems.00799-22-s0001.pdf]

Table S1. The intra- and inter-clone SNP numbers of ST11 CRKP strains

SNP	A	**B**	**C**	**D**	E	**F**	**G**	**H**	**I**	**J**	**K**	**L**	**M**	N
A	-	81-84	70-72	65-67	76-76	49-53	95-98	69-71	91-97	68-70	100-106	131-131	69-70	97-97
**B**		2-5	51-55	48-53	60-63	60-67	100-105	48-54	97-106	52-57	106-114	136-138	49-53	104-107
**C**			1-1	37-41	50-52	49-56	90-95	23-27	85-93	43-47	96-104	125-126	41-43	91-93
**D**				0-4	43-45	44-51	86-91	37-41	82-90	35-39	92-100	125-127	38-41	88-90
E					-	57-62	95-98	50-52	95-101	48-50	101-107	134-134	51-52	100-100
**F**						0-6	77-84	49-56	72-81	49-56	81-91	111-114	49-54	79-83
**G**							0-4	91-96	101-110	91-96	53-62	80-83	91-94	107-110
**H**								0-4	87-95	42-46	96-104	127-130	38-42	94-96
**I**									0-13	87-95	106-118	134-140	85-92	89-95
**J**										0-4	96-104	129-131	43-46	92-94
**K**											1-4	89-95	95-102	111-117
**L**												0-0	127-127	141-141
**M**													0-1	94-95
N														-

For clones comprising two or more isolates, which are in bold, the minimum and maximum numbers of SNP are shown.
